# Conjunctival granuloma with necrosis associated with exposed suture in upper double lid masquerading as ocular surface squamous neoplasia: a case report

**DOI:** 10.1186/s12886-017-0457-8

**Published:** 2017-04-26

**Authors:** Yu Jeong Kim, Jaeyoung Kim, Hokyung Choung, Mee Kum Kim, Won Ryang Wee

**Affiliations:** 1 Department of Ophthalmology, Seoul National University College of Medicine, Seoul, Korea; 2 Laboratory of Ocular Regenerative Medicine and Immunology, Seoul Artificial Eye Center, Seoul National University Hospital Biomedical Research Institute, Seoul, Korea; 3 Department of Ophthalmology, Seoul Metropolitan Government Seoul National University Boramae Hospital, Seoul, Korea; 4 Department of Ophthalmology, Seoul National University Hospital, 101 Daehak-ro, Jongno-gu, 110-744 Seoul, Korea

**Keywords:** Conjunctival mass, Conjunctival granuloma, Upper blepharoplasty, Exposed suture, Squamous cell carcinoma

## Abstract

**Background:**

This study reports two cases of conjunctival granuloma with necrosis caused by an exposed suture in the upper palpebral conjunctiva masquerading as ocular surface squamous neoplasia.

**Case presentation:**

Two patients presented with chronic conjunctival ulcerative and granulomatous lesions on the superior bulbar conjunctiva that repeatedly recurred after the mass was removed. The pathologic findings revealed the absence of malignant cells and presence of many lymphocytes, plasma cells, and histiocytes. There was no evidence of acid-fast bacilli or fungal organisms. When a past history of blepharoplasty was established, microscopic examination revealed occult exposed suture tips. After the sutures were removed, the granuloma with necrosis was resolved within a month.

**Conclusion:**

For all conjunctival lesions in the superior bulbar conjunctiva, a thorough examination of the ocular adnexae which includes eyelid eversion should be performed. There should be a suspicion of foreign body or exposed suture material especially when there is a non-healing ulcer.

## Background

Ocular surface squamous neoplasia (OSSN) of the conjunctiva is considered the most common malignancy of the ocular surface [1, 2]. Because of the potential invasive and sometimes aggressive properties of OSSN [3], clinical suspicion of OSSN is critical for timely treatment. The presence of feeder vessels, necrotic lesions, or nodular lesions raises the suspicion of malignancy including OSSN [3]. The differential diagnoses of a conjunctival lesion, which can masquerade as OSSN, include foreign body reaction, necrotizing scleritis, infectious conjunctivitis, chemical burn, conjunctival lymphoma, conjunctival melanoma, limbal dermoid, pterygium, pinguecula, and amyloidosis [4, 5].

Meanwhile, the complications associated with exposed sutures following upper blepharoplasty are vertical cornea abrasions, eyelid edema, papillary changes in the upper tarsal conjunctiva, and recurrent blepharoptosis [6]. As far as we know, there is a no report of necrotic granuloma due to exposed suture on the bulbar conjunctiva. Herein we report two cases of upper conjunctival necrotic nodular lesions caused by retained sutures masquerading conjunctival malignancy that followed upper blepharoplasty.

## Case presentation

### Case 1

A 43-year-old woman in good health was referred for evaluation of a superior bulbar conjunctival necrotic lesion in the left eye. She presented with ocular pain and a foreign body sensation in the left eye that had lasted for 1 year. She said that she had no history of trauma or surgery.

**Fig. 1:**
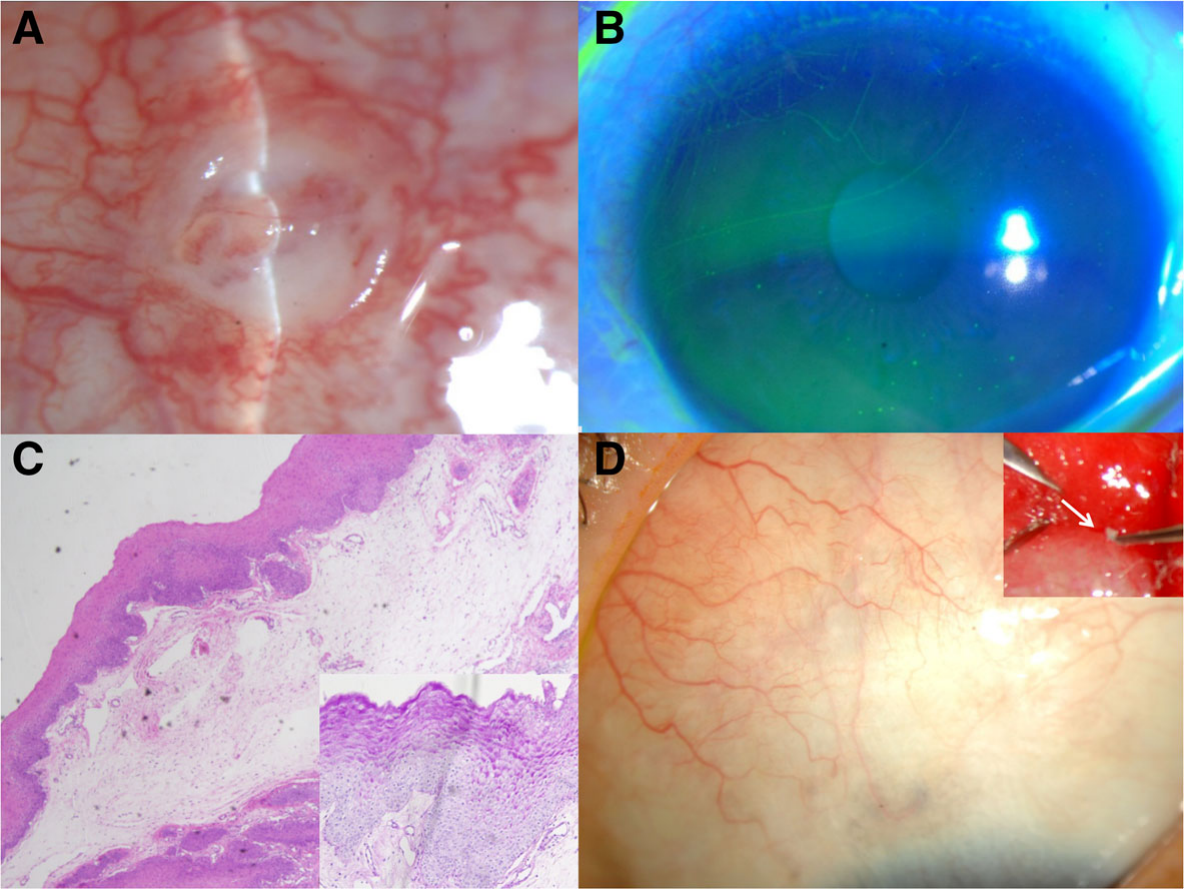
**a** An elevated bulbar conjunctival mass, measuring 2.2 mm, had a central ulceration with granuloma. The surrounding conjunctiva was moderately injected. The lesion resembled a conjunctival malignancy. **b** There were some linear epithelial abrasions on the superior cornea. **c** Histopathologic examination revealed conjunctiva with epithelial hyperplasia, chronic inflammatory infiltrate consisting of lymphocytes, plasma cells, and histiocytes. (hematoxylin and eosin; original magnification ×4). **d** Exposed suture fragment was detected near the cul-de-sac and removed under local anesthesia (white arrow). The lesion was completely healed within 1 month

The slit-lamp examination revealed moderate injection and a 2.2 mm round ulcerated zone with granuloma in the center of the superior bulbar conjunctiva (Fig. 1a). There were linear epithelial abrasions on the superior cornea that were suspected to be from a foreign body in the upper palpebral conjunctiva (Fig. 1b), but a gross search observed only a severe papillary reaction in the everted lid. The rest of her ophthalmological examination was normal.

Conjunctival culture and excisional biopsy were conducted with stains including Gram, Gomori’s methenamine silver (GMS), Periodic acid–Schiff (PAS), and acid-fast bacilli (AFB) to rule out mycobacterial conjunctivitis or conjunctival malignancy. Systemic evaluations revealed no abnormal findings. AFB staining revealed no acid-fast bacilli, and GMS and PAS staining revealed no fungal organisms, although *Bacillus* species were cultured. After topical antibiotics that treated Gram-positive bacteria were administered, conjunctival necrosis decreased, but the granulomatous lesion remained. The pathologic findings showed CD3+ T cells, CD20+ B cells, CD68+ macrophages, and Ki-67+ positive cells in 1% of the parabasal area, which was diagnosed as inflammatory conjunctivitis, but there was no evidence of malignancy (Fig. 1c). To reduce inflammation, systemic and topical immunosuppression treatment was initiated, but the conjunctival lesion persisted. We thoroughly checked the past history, and found a history of blepharoplasty many years earlier. Therefore, we explored her tarsal conjunctiva in the operating room with a surgical microscope and detected a pinpoint dot near the cul-de-sac that was revealed to be occult exposure of the suture tip (Fig. 1d). After removal of the entire buried suture knot, the necrotic granuloma was completely resolved (Fig. 1d).

### Case 2

**Fig. 2:**
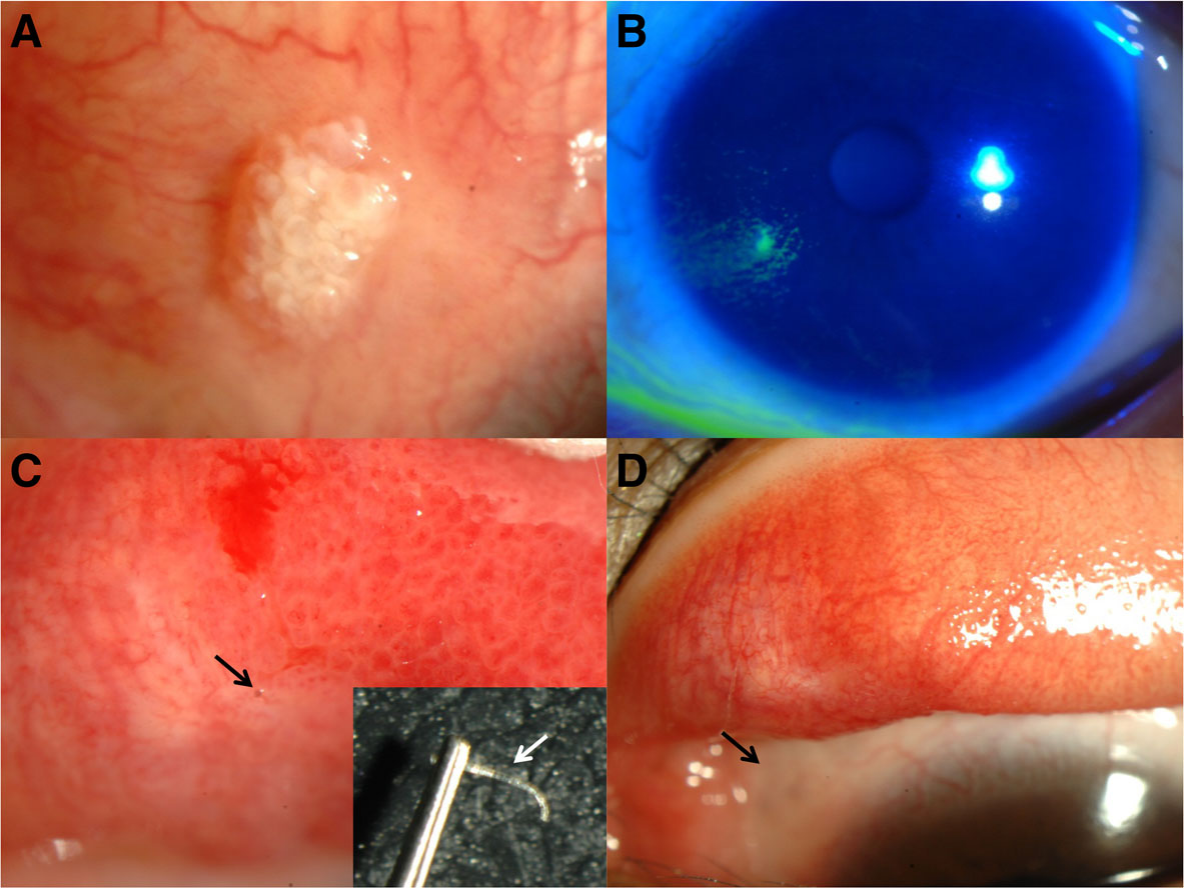
**a** A nodulated mass with calcium depositions was located on superior bulbar conjunctiva. **b** The cornea was relatively clear with no epithelial defects. **c** With eversion of the superior conjunctival fornix, a nonabsorbable suture with focal papillary reaction was detected on superior palpebral conjunctiva (*black arrow*). The suture was removed with slit lamp (white arrow). **d** After 2 months, the lesion was resolved (*black arrow*)

A 25-year-old woman was referred for evaluation of a superotemporal bulbar conjunctival mass in the left eye. At a previous ophthalmic clinic, she had complained of a foreign body sensation. On suspicion of OSSN, a conjunctival biopsy was performed that revealed no malignant (Ki-67+) cells but an inflammatory lesion that contained CD3+ T cells and CD20+ B cells. She was treated with a topical steroid and topical antibiotics for 8 months, but the lesion persisted. When the patient was referred to our clinic, a slit-lamp examination revealed a granulomatous mass and calcium deposits in the center of a granuloma along with vascular injection on the superior bulbar conjunctiva (Fig. 2a). The cornea was relatively clear with no epithelial defects (Fig. 2b). We asked about a history of blepharoptosis surgery, and the patient had had upper blepharoplasty 5 years earlier. With eversion of the upper lid, diffuse papillary reaction and an exposed nonabsorbable suture were detected on the superior palpebral conjunctiva near the medial canthal area of the fornix (Fig. 2c). After the suture was removed (Fig. 2c), the granulomatous mass with calcific deposits was completely resolved (Fig. 2d).

## Discussion

In various Far East Asian countries, double-eyelid blepharoplasty is a popular aesthetic surgery. A current popular method in Far East Asian countries is non-incision buried suture blepharoplasty because of cosmetic advantages, short operative and recovery times. Surgical technique of non-incision blepharoplasty is as follows. After turning the upper eyelid inside out, 7–0 nylon suture is passed through the same point through the upper tarsus margin from the conjunctiva to the skin [7, 8]. In the patients with blepharoptosis, this suture is introduced into the upper eyelid through the conjunctiva close to the superior fornix [9]. This suture is then threaded in one direction through the skin and upper part of the tarsus alternately through each of the point incisions. At the medial end, the suture is passed through the subcutaneous tissue to the lateral end. The knot is tied by multiple-knottings to reduce loosening and is buried within the lateral stab incision. By tightening the threads, the superior palpebral levator muscle and the tarsus are connected and the tarsus is elevated. Its only associated complications were reported to be foreign body sensation, hematoma on eyelid and palpebral subconjuctival inclusion cyst [9, 10]. In these reports, we describe unusual complications such as granulomatous foreign body reactions on the superior bulbar conjunctiva following blepharoptosis surgery that could have been misdiagnosed as OSSN or tuberculous granuloma.

Usually, foreign bodies or exposed sutures on the palpebral conjunctiva of the upper lid cause vertical abrasions on the cornea, and foreign body-associated corneal lesions are easily diagnosed by this observation. However, our patients presented with conjunctival necrosis and granuloma, which were very unusual findings for exposed sutures on the upper lid. One case did not even feature cornea abrasions. The reason for this unique conjunctival presentation may be presumably related to the location of the exposed or broken suture; deep-seated sutures near the fornix appear to irritate the conjunctiva more than the cornea. The reason of the deep seated location of the exposed or broken sutures is that the loosened sutures may be migrated to the deep fornix or the sutures may be placed on superior fornix if the patients had blepharoptosis component. Because of the deep location near the fornix, the suture appeared to be less mobile despite the blinking movement of the lid, and the tip of the suture appeared to persistently irritate the corresponding area of the upper conjunctiva where the suture made contact. This could cause necrosis and granulomatous inflammation rather than epithelial linear abrasions.

After any mechanical injury, various inflammatory responses are activated with many types of cells including immune cells (neutrophils, monocytes, lymphocytes and dendritic cells), endothelial cells, keratinocytes and fibroblasts [11]. Histology of the 1st case was performed in our hospital. To rule out the malignancy, we stained the tissue using anti-Ki67 for highly proliferating cells, and it revealed no suspicion of malignancy (1% of parabasal area of the conjunctiva). To rule out the inflammation, we stained the tissue using anti-CD3 for T cells, anti-CD20 for B cells and anti-CD68 for macrophage. Therefore, we found T cells, B, cells and macrophages were involved in this chronic inflammation and wound healing process. However, in 2nd case, the histology was conducted in secondary hospital before referral to us. They stained the tissue using anti-CD3 for T cells, anti-CD20 for B cells and anti-Ki67 for malignant cells. They did not use anti-CD68 for macrophages. Although we do not know the involvement of macrophages in the tissue exactly, involvement of T and B cells in the 2nd case were similar to those in the 1st case. Ki67^**+**^ cells were not observed as same as the 1st case. Considering that the granulomatous changes was observed in 2nd case, we can presume that macrophages might also be involved in the chronic wound healing process in the 2nd case. Taken together, the histologic features are similar in two cases, showing chronic inflammation.

Necrotic granulomatous lesions resemble OSSN or tuberculous conjunctivitis and thus require appropriate suspicion of OSSN or infectious conjunctivitis. No involvement of the limbus and relative young age may lower the possibility of malignancy, and no systemic involvement of tuberculosis may reduce the possibility of tuberculous conjunctivitis. Taken together, in a young patient who has undergone upper blepharoplasty, chronic necrotic granulomatous conjunctival lesions in the superior bulbar area should be considered possible suture-related inflammation as well as possible OSSN or infectious conjunctivitis. Very careful examination of the palpebral conjunctiva through the fornix may ward against unnecessary biopsies of suture-related granulomas.

## Conclusion

Blepharoplasty for cosmetic reasons has grown more prevalent in Asian countries. Thus, surgeons in Asian countries should be aware of this blepharoplasty-related complication, which can masquerade as OSSN or infectious conjunctivitis.

## Abbreviations

AFB: Acid-fast bacilli
GMS: Gomori’s methenamine silver
OSSN: Ocular surface squamous cell neoplasm;
PAS: Periodic acid–Schiff
